# State-of-the-art and prospects for intense red radiation from core–shell InGaN/GaN nanorods

**DOI:** 10.1038/s41598-020-76042-0

**Published:** 2020-11-04

**Authors:** Evgenii A. Evropeitsev, Dmitrii R. Kazanov, Yoann Robin, Alexander N. Smirnov, Ilya A. Eliseyev, Valery Yu. Davydov, Alexey A. Toropov, Shugo Nitta, Tatiana V. Shubina, Hiroshi Amano

**Affiliations:** 1 Ioffe Institute, 26 Politekhnicheskaya, St Petersburg, Russia 194021; 2 Institute of Materials and Systems for Sustainability (IMaSS), Nagoya University, Nagoya, Japan

**Keywords:** Optical spectroscopy, Nanoscale materials

## Abstract

Core–shell nanorods (NRs) with InGaN/GaN quantum wells (QWs) are promising for monolithic white light-emitting diodes and multi-color displays. Such applications, however, are still a challenge because intensity of the red band is too weak compared with blue and green. To clarify this problem, we measured photoluminescence of different NRs, depending on power and temperature, as well as with time resolution. These studies have shown that dominant emission bands come from nonpolar and semipolar QWs, while a broad yellow-red band arises mainly from defects in the GaN core. An emission from polar QWs located at the NR tip is indistinguishable against the background of defect-related luminescence. Our calculations of electromagnetic field distribution inside the NRs show a low density of photon states at the tip, which additionally suppresses the radiation of polar QWs. We propose placing polar QWs inside a cylindrical part of the core, where the density of photon states is higher and the well area is much larger. Such a hybrid design, in which the excess of blue radiation from shell QWs is converted to red radiation in core wells, can help solve the urgent problem of red light for many applications of NRs.

## Introduction

Core–shell nanorods (NRs), which comprise InGaN quantum wells (QWs) in a shell deposited over a GaN core, are among the most promising objects of modern nanophotonics^1^. It is assumed that monolithic NR-based devices can be used in multi-color displays and solid-state lighting^2,3^ instead of commonly used phosphor-coated light emission diodes (LEDs)^4^. The InGaN/GaN QWs are a good basis for that, because they can emit light in any part of the visible range depending on QW parameters such as thickness and composition^5,6^. In the core–shell NRs, the variation of these parameters occurs spontaneously as a result of difference in both the growth rate and in the incorporation of indium for different crystallographic planes.

Actually, there are many obstacles to achieve these aims. To get white light, for instance, one should combine the light of two (Y, B) or three (R, G, B) wavelengths with comparable intensities. However, the internal quantum efficiency (IQE) of InGaN/GaN QWs is usually low in the yellow-red spectral range^7^. The InGaN radiation gets into this range when the well width is large and In content is $x\simeq 0.4$-0.6. When the In content is so high, the critical thickness diminishes and the phase separation takes place that inevitably deteriorates the QW quality^8^. In addition, the built-in electric field across the polar or semipolar QWs spatially separates electrons and holes decreasing the IQE^9^. The overlap of electron and hole wave functions cannot exceed 1% in polar InGaN/GaN QWs emitting at wavelengths of more than $\sim 560$-600 nm^10^. The quantum-confined Stark effect (QCSE) is stronger in the wide InGaN/GaN QWs with high In content due to the increasing strain and concomitant piezoelectric polarization. However, a positive feature of the QCSE is the shift of the emission towards the longer wavelengths that sometimes is crucial for obtaining red emission.

Over the past two decades, various approaches to the manufacture of monolithic InGaN/GaN white LEDs have been reported. In several papers, the blue light emitted from an electrically pumped active region was partially converted into yellow-red photoluminescence (PL) by the QWs of different widths and compositions. More importantly, these QWs, situated outside the active region, were pumped only optically^11–15^. This approach makes it possible to compensate for the small efficiency of red radiation by increasing the number of the re-emitting QWs. An alternative approach implies that the active region includes InGaN inserts (quantum wells, disks, or dots) of various width/composition/polarity designed to jointly cover the entire visible range. This approach dominates up to now^3,16–24^. It is worth mentioning that the increase in the number of QWs when all of them are pumped electrically does not mean a proportional increase in electroluminescence (EL) intensity because of the limitation of carrier injection and transport^25^.

The state-of-the-art core–shell NRs are usually designed in accordance with the last approach. Polar, semipolar, and nonpolar QWs, formed simultaneously, can emit in the red, green, and UV-blue regions, respectively^3,26^. The advantages of such three-dimensional (3D) structures over planar ones are numerous: low dislocation density, use of nonpolar planes, large effective surface area, and high light extraction efficiency^1^. As a rule, the polar QWs at the top of NRs are wider and have higher content of indium than semi- and nonpolar QWs. Together with the strong QCSE in the polar QWs, this shifts the radiation towards longer wavelengths^2,27,28^. Systematically, a weak red EL signal appears first at low applied voltage; when voltage increases, the blue/green radiation begins to dominate^2,26^. The placement in the NR tip is not very successful for polar QWs: their area is usually much smaller than that of nonpolar wells, and as a result, the radiation intensity is low. Generally, the red radiation can be enhanced by using truncated pyramids^24^ or by increasing the NR diameter^28^. In this case, however, important advantages of the NRs such as dislocation filtration and large effective area can be lost and the IQE will fall^29^.

**Figure 1:**
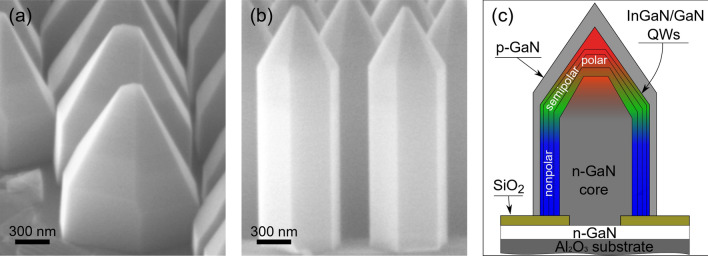
(**a**, **b**) Scanning electron microscopy images of two NR samples: A1 (**a**) and A2 (**b**); (**c**) sketch of the core–shell GaN/InGaN nanorod.

In this paper, we report on comparative studies of core–shell NRs and planar samples with InGaN/GaN QWs, as well as NRs without any wells. PL spectroscopy, including time-resolved PL (TRPL), performed over a wide temperature range from low temperature (LT) of 10 K to room temperature (RT), and micro-PL ($\mu$-PL), performed at different excitation wavelenghts and powers, allows us to attribute the observed two dominant PL peaks to nonpolar and semipolar QWs. We show how these peaks shift from UV to green spectral regions depending on the nominal QW composition, and demonstrate their relatively high IQE. In contrast, the radiation from polar QWs is very weak. The broad yellow-red band previously attributed to these wells^26^ appears to be mainly associated with defects in the GaN core of nanorod. To further clarify the current situation, we present the calculations of electromagnetic field distribution in NRs, which shows a negligible density of photon states at NR tips. This can lead to additional suppression of red radiation, which was initially weak due to the small area of the polar QW and inherent QCSE. As a way to solve these problems, we consider the hybrid design of NRs, where the polar QWs of a larger area are located in the core, being excited by blue radiation of nonpolar wells.

## Experimental results

### Samples description

We study three sample sets: (i) NRs of large diameter (A1–C1), (ii) NRs of small diameter (A2–C2) and (iii) planar samples (A–C). All samples contain three InGaN/GaN QWs situated in the GaN p-n junction. Each set includes three samples which differ by In composition in QWs that is realized by the variation of the growth temperature, ${\mathrm{T}}_{\mathrm{gr}}$ (see Table 1 in Methods). Details of their fabrication are given in Methods. An additional reference sample is an array of undoped GaN NRs without any QWs. Scanning electron microscopy (SEM) reveals that typical NRs A1, B1, and C1 have both height and diameter of $\sim$ 1.3 $\mu \hbox {m}$, while NRs A2–C2 are about 780 nm in diameter and 2 $\mu \hbox {m}$ in height. Figure 1 shows representative SEM images of the NRs A1 (a) and A2 (b), as well as a schematic design of the NRs (c). For NRs A1–C1, the areas of the vertical ($10\bar{1}0$) sidewalls and the pyramidal ($10\bar{1}1$) facets are comparable, while for NRs A2–C2 the pyramidal ($10\bar{1}1$) facets are noticeably smaller. The (0001) facets are very small in all studied NRs.

### Luminescence related to defects in GaN

**Figure 2:**
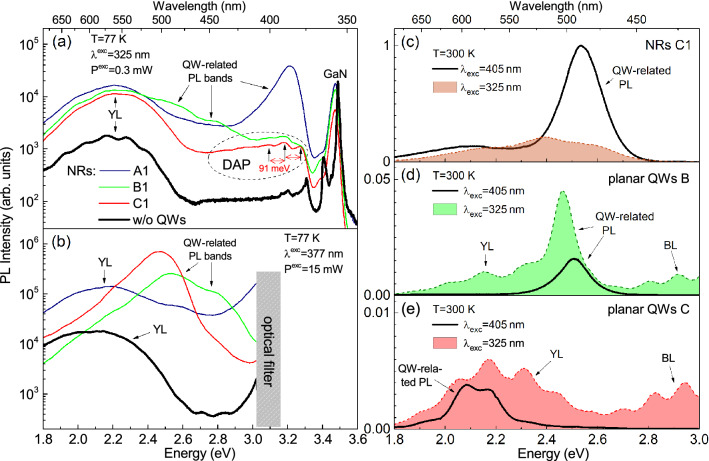
(**a**, **b**) Macro-PL spectra of the NR arrays A1, B1, and C1, measured at $\hbox {T}=77$ K under above-barrier excitation ($\lambda _{{\mathrm{exc}}}=325$ nm, $\hbox {P}_{{\mathrm{exc}}}=0.3$ mW) (**a**) and under-barrier one ($\lambda _{\mathrm{exc}}=377$ nm, $\hbox {P}_{\mathrm{exc}}=15$ mW) (**b**). Thick black lines in (**a**) and (**b**) show spectra of NRs without any QWs; (c–e) $\mu$-PL spectra of NRs C1 (**c**) with maximal In content and two planar QWs—B (**d**) and C (**e**), measured at RT with excitation wavelength $\lambda _{\mathrm{exc}}=405$ nm ($\hbox {P}_{\mathrm{exc}}=160$ $\mu \hbox {W}$, solid lines) and 325 nm ($\hbox {P}_{\mathrm{exc}}=140$ $\mu \hbox {W}$, dashed lines, filled area).

PL bands related to defects in GaN can be recognized by their characteristics such as spectral shape, peak energy, decay time, as well as by the behavior of its intensity with the excitation power and temperature variation. Although the nature of the defects responsible for the specific PL bands is still debated^30^, their PL properties are detailed in the literature ^31^. In particular, the yellow luminescence (YL) band has a nearly Gaussian shape with a peak at 2.2–2.3 eV and the full width at half maximum (FWHM) of about 400 meV. This band is attributed to the optical transition between the conduction band (or shallow donor) and deep acceptor with an activation energy of 0.8–0.9 eV. With increasing temperature, the YL is relatively stable in intensity up to 450 K, and its spectral position does not change despite the GaN band-gap reduction. These features are explained within the framework of the configuration coordinate model for a deep acceptor with strong electron-phonon interaction. The so-called blue luminescence (BL) band, situated around 2.9 eV, is explained by similar transitions but with the participation of a deep acceptor with an activation energy of 0.34-0.4 eV. Another defect-related PL band, called either as donor-acceptor pairs luminescence (DAP) ^32^, or ultraviolet luminescence (UVL) ^31^, can be distinguished from QW-related PL by its specific shape with a maximum around 3.25 eV and several phonon replicas, separated by the longitudinal-optical phonon energy of 91 meV (see Fig. 2a). This band is associated with optical transitions between the shallow donor and shallow acceptor level. PL of point defects in GaN usually decays in the microsecond time domain, which is determined by the distance between donors and acceptors^32^. Due to long decay times and limited density of defects, the intensity of such PL bands increases according to either saturating or sub-linear law with increasing pump power^31^, which makes it possible to differentiate between defect-related and QW-related PL. The latter usually has an approximately linear or super-linear dependence of the intensity on the $\hbox {P}_{\mathrm{exc}}$ in the same ranges^33,34^.

The samples were studied by both $\mu$-PL and macro-PL as described in the Methods. Figure 2a shows macro-PL spectra of NRs A1, B1, and C1, as well as of reference NRs without QWs, measured at 77 K in the regime of above-barrier excitation with relatively small power, which was most suitable for detection of the defect-related PL. The spectra contain a broad yellow-red PL band with a peak wavelength of 560-600 nm. Broad luminescence bands in the yellow-red spectral region are often attributed to the radiation of polar QWs in the core–shell NRs^2,27,28^. However, in our case, its shape does not depend on the nominal In content. In addition, its shape is similar to the spectrum of the YL band, measured in the NRs without QWs. Such features indicate a significant contribution of defect-related states in the GaN core to this yellow-red radiation, as noted in our previous work^35^. The defect-related DAP band is also present in the spectra measured at low temperatures. For NRs A1, its phonon replicas are overlapped with the QW-related PL at 385 nm. The spectra of NRs B1 and C1, obtained with under-barrier excitation at 77 K, dominate by the QW-related PL (Fig. 2b). One can see that this PL band shifts from the near-UV region (Fig. 2a, sample A1) to blue and green ones (Fig. 2b, samples B1 and C1) with an increase in the nominal In content.

Figure 2c–e shows the $\mu$-PL spectra of NR sample C1 (c) and two planar samples—B (d) and C (e)—for the cases of the under-barrier (405 nm) and above-barrier (325 nm) excitation. In this regime, a higher excitation power density is realized in comparison to Fig. 2a,b, which facilitates the detection of PL bands associated with QWs against the background of a saturated band associated with defects. The PL bands of NRs are blue-shifted and have higher intensity than in the planar QWs of the same nominal In content. Certainly, the NRs provide an apparent gain in PL intensity at RT in the blue-green region around $\sim 500$ nm. Another PL peculiarity follows from a comparison of the PL spectra obtained upon above-barrier and under-barrier excitations. For planar samples, the main difference is the amplification of the YL upon the above-barrier excitation. The above-barrier excitation of a QW is usually more effective due to the enhanced generation of carriers in a thick barrier. This situation is realized in the case of the planar sample B (Fig. 2d). However, for the planar sample C, the QW-related PL emission at RT was detected with under-barrier excitation only (Fig. 2e). This means that the supply of carriers from the barrier to the QWs is less efficient than the resonant excitation of carriers in the QWs in this sample. For NRs C1, the $\mu$-PL spectrum turns out to be $\sim 5$ times more intense for the under-barrier excitation compared with the above-barrier one (Fig. 2c). The shape of the spectra significantly depends on the excitation wavelength ($\lambda _{\mathrm{exc}}$): the blue-green radiation of the QWs dominates for under-barrier excitation ($\lambda _{\mathrm{exc}}=405$ nm) and the wide yellow-red and green bands dominate for $\lambda _{\mathrm{exc}}=325$ nm. Apparently, the carrier capture by defect states in GaN suppresses other recombination channels. Thus, the question arises whether or not at least some part of the yellow-red PL band is related to QWs located in the NR shell.

**Figure 3:**
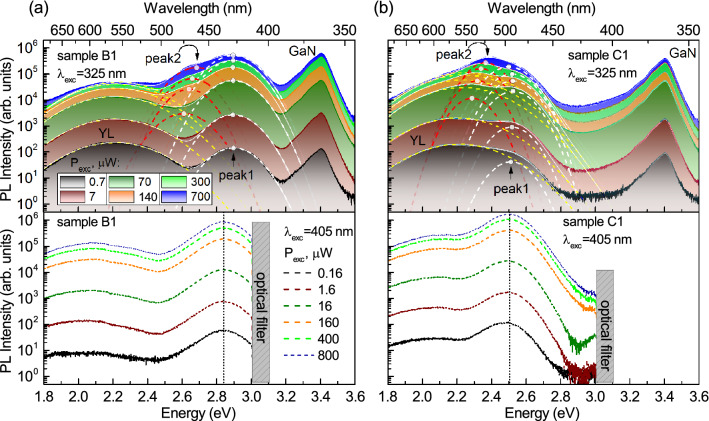
Power-dependent $\mu$-PL spectra measured at RT in the NR samples B1 (**a**) and C1 (**b**) excited by cw-laser lines with wavelengths of 325 nm (top panel) and 405 nm (bottom panel). The yellow, white and red dashed lines show the fitted Gaussian functions of the YL band, peak 1 and peak 2, respectively, while the white solid lines show their sum.

**Figure 4:**
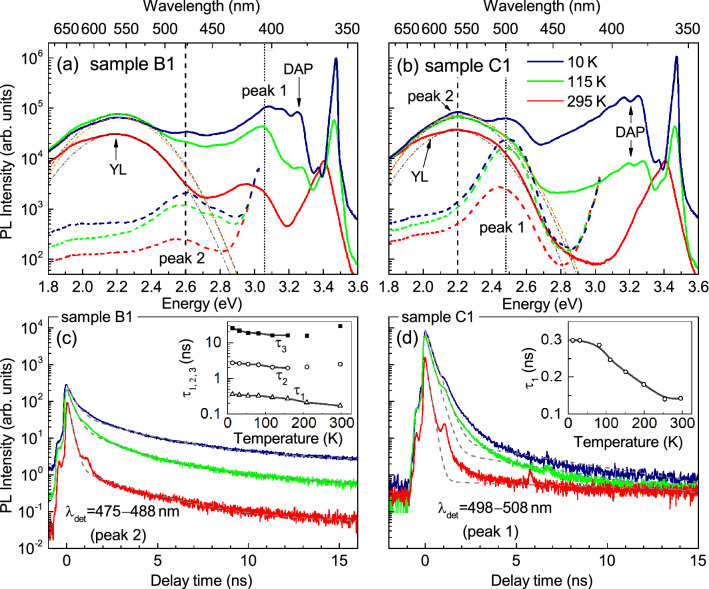
Temperature-dependent PL spectra (**a**, **b**) and PL decay curves (**c**, **d**) measured in NR arrays B1 (**a**, **c**) and C1 (**b**, **d**). The PL spectra were obtained with an excitation wavelength of 325 nm (solid lines, P_exc_ = 5 mW) or 405 nm (dashed lines, P_exc_ = 0.4 mW). The decay curves were detected at a wavelength of the PL peak excited by laser line 405 nm with average power of 0.4 mW and repetition rate of 30 MHz. Dash-dotted lines in (**a**, **b**) show Gaussian functions with a position and FWHM of about 2.2 eV and 420 meV, respectively, which represents the YL band. In (**c**, **d**), the dashed curves are fitting functions, and background level is $4.6\cdot 10^{-4}$. The sharp peaks around the main one, as well as around the time of 6 ns (in (**d**)), are apparatus. Zero time is taken at the decay curve maxima. The inserts in (**c**, **d**) show temperature dependences of the decay time constants $\tau _{\mathrm{i}}$.

**Figure 5:**
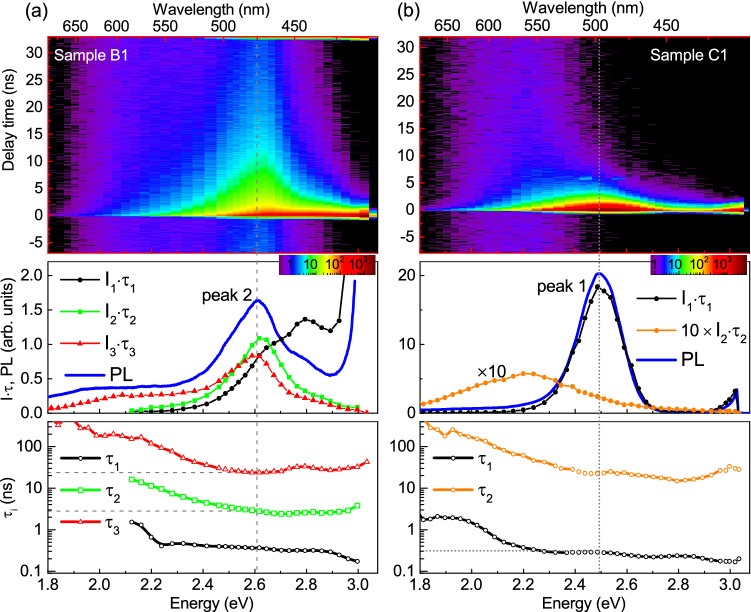
TRPL plots of the PL intensity (in logarithm scale marked by color) measured in NR arrays B1 (**a**) and C1 (**b**) at 15 K (top panels). Bottom panels show the spectral variation of characteristic decay times $\tau _{\mathrm{i}}$ extracted from the PL decay curves for these samples. Corresponding spectra of PL (measured by CCD) and ${\mathrm{I}}_{\mathrm{i}}\cdot \tau _{\mathrm{i}}$ are shown in the middle panels.

### Power dependences of micro-photoluminescence in the NRs

To detail the origin of the observed PL bands, we performed power-dependent measurements of $\mu$-PL with excitations above and below the barrier. Typical series of RT $\mu$-PL spectra measured in the single NRs B1 and C1 are shown in Fig. 3a,b. The corresponding spectra of NRs A1 are shown in Fig. S2 in the Supplementary. The spectra were approximated by Gaussian functions as described in the Methods, and the intensities, spectral positions, and spectral widths of the main peaks are shown in Fig. S3 in the Supplementary. For the excitation wavelength of 325 nm, the spectrally broad yellow-red band dominates at the weak $\hbox {P}_{\mathrm{exc}}=0.7$ $\mu \hbox {W}$. However, its intensity tends to saturation with increasing $\hbox {P}_{\mathrm{exc}}$ (see Fig. S3 in the Supplementary). We observed similar variation in the PL spectra of planar samples (see Fig. S5 in the Supplementary). This behavior allows us to finally conclude that the yellow-red PL upon excitation above the barrier is mainly associated with point defects in GaN. The $\mu$-PL spectra obtained with $\lambda _{\mathrm{exc}}=325$ nm contain also peak 1, whose wavelength is 390, 425, and 490 nm in NR arrays A1, B1, and C1, respectively. As P$_{\mathrm{exc}}$ increases, another peak 2 appears at the wavelengths of $\sim$ 470, 485, and 540 nm, respectively (Fig. 3). The peak 1 exists even at small powers (0.7 $\mu \hbox {W}$) and its spectral position remains almost unchanged with increasing $\hbox {P}_{\mathrm{exc}}$ to 700 $\mu \hbox {W}$. The fitting procedure reveals that the peak 2 shifts towards higher energy by approximately 173, 75, and 79 meV in NR arrays A1, B1, and C1, respectively (see Fig. S3 in the Supplementary). The intensity of the peak 2 and the value of the energy shift vary among the rods of the same sample, whereas the intensity of the peak 1 is more stable. With the under-barrier excitation, the peak 2 disappears (Fig. 3, bottom panel) or decreases significantly (Fig. 4a). The $\mu$-PL spectra, measured in NRs of a smaller diameter (samples A2, B2, and C2) have similar features. There are some differences from the PL spectra of NRs with large diameter, e.g., the reduced intensity of the peak 2, as can be seen in Fig. S4 in the Supplementary.

### Temperature-dependent and time-resolved photoluminescence

The temperature dependencies of macro-PL spectra measured in NR arrays B1 and C1 are shown in Fig. 4a,b. The spectra substantially depend on $\lambda _{\mathrm{exc}}$. For instance, in sample B1 the peak 1 appears at 420 nm upon above-barrier excitation (solid curves), while the weak peak 2 appears at 475 nm upon under-barrier excitation (dashed curves) at LT. Nevertheless, the PL intensity ratio $\hbox {I}(300~\hbox {K})/\hbox {I}(10~\hbox {K})$ for both peaks is in the 12-15 % range ($\lambda _{\mathrm{exc}}=377$ nm, $\hbox {P}_{\mathrm{exc}}=15$ mW). For the planar samples, this ratio is smaller than 1 %, and TRPL reveals that nonradiative processes dominate at RT (see Fig. S6 in the Supplementary).

We performed the low-temperature TRPL studies with under-barrier excitation, which is more appropriate to reveal the radiation of QWs. We will focus on two samples with highest nominal In content (B1: x = 0.19 and C1: x = 0.26). The dependencies of PL intensity on time and energy are shown in the top panels of Figure 5a,b for the NR arrays B1 and C1, respectively. They exhibit the presence of both rapidly and slowly decaying components. In order to estimate their particular contributions, the decay curves were approximated by the sum of two or three exponential functions

1 $$\begin{aligned} I(t) =\sum _{i=1}^{} I_i e^{-(t-t_0)/\tau _i}, \end{aligned}$$

where $t_0$ is the excitation time and $\tau _i$ is the $i^{th}$ decay time constant. Spectral dependencies of $\tau _i$, obtained for NR arrays B1 and C1, are shown in the bottom panels of Fig. 5. The value of $\hbox {I}_i\cdot \tau _i$ reflects the contribution of $i^{th}$ component to the total PL signal, since it equals to the integral of $i^{th}$ term in Eq. (1) in the range from $t_0$ to infinity. For sample B1, the dominant contribution to the peak 2 is represented by two slowly decaying components with decay times $\tau _2\approx 3$ ns and $\tau _3\approx 25$ ns, as can be seen from the comparison of PL spectrum measured by charge-coupled detector (CCD) and $\hbox {I}_i\cdot \tau _i$ spectra in the middle panel of Fig. 5a. On the contrary, for sample C1 only the fast decaying component ($\tau _1\approx 0.3$ ns) contributes to the peak 1. The weak low-energy shoulders of the peak 1 and peak 2 have characteristic decay times of the order of 100 ns.

Figure 4c,d shows the temperature-dependent PL kinetics, measured at NR arrays B1 and C1. The detection wavelength was set to the maximum of the peak 2 (475-488 nm) for sample B1 and to the maximum of the peak 1 (498-508 nm) for NRs C1. The decay time $\tau _1$ is equal to 0.36 and 0.3 ns at LT for samples B1 and C1, respectively. With temperature rising, $\tau _1$ decreases down to 0.14-0.15 ns at RT (see the inserts in Fig. 4c,d), which is close to the time resolution of our setup. For the PL peak 1 of sample C1, we can suggest that the LT value of $\tau _1\approx 0.3$ ns is close to the radiative time $\tau ^{rad}$ of these transitions, since the fast decaying PL component controls the peak 1 and changes little with temperature rise up to 80 K. For the peak 2 of sample B1, the PL decay is defined by the multi-exponential function with characteristic decay time constants $\tau _2\approx 3$ ns and $\tau _3\approx 25$ ns at LT, which complicates the evaluation of $\tau ^{rad}$. The temperature dependencies of $\tau _{1-3}$ measured in the peak 2 indicate that the nonradiative recombination processes are significant even at LT for these transitions.

## Discussion and prospects

The direct method for determining the spatial origin of various PL bands in core–shell NRs is cathodoluminescence^27,36^. Besides, the place of occurrence of a PL band in NRs can also be determined by analyzing their radiative characteristics, since they differ for polar, semipolar, and nonpolar QWs. Indeed, the presence of a built-in electrostatic field in polar and semipolar QWs leads to spatial separation of electrons and holes. This results in a low-energy shift of the corresponding luminescence band and to an increase in the radiative recombination time in comparison with the similar QWs without the built-in field. Typical PL decay times for polar QWs on the *c*-plane are tens of nanoseconds, while for semipolar and nonpolar QWs they are units of nanoseconds and hundreds of picoseconds, respectively^37,38^. TRPL studies also reveal that PL often decays according to a nonexponential law in polar and semipolar QWs, while nonpolar QWs often exhibit a one-exponential decay^39,40^. Possible reasons for the non-exponential decay of the PL is the independent localization of electrons and holes, which occurs due to the built-in field and fluctuations of the QW potential associated with the inhomogeneity of the composition and thickness, while the exponential decay of PL in nonpolar QWs can be due to exciton luminescence^40^. With an increase in the pump power density, a high-energy shift of the PL bands in polar and semipolar QW occurs^41–43^, which is small or absent for nonpolar QWs^43^. This shift can be explained by a combination of two processes—screening of the built-in electrostatic field by mobile charge carriers and overflow of the localized states^44–46^.

These characteristic features allow us to differentiate the contributions to the PL spectra from different types of QWs—nonpolar, semipolar, and polar. The RT spectra contain peaks 1, 2, and a wide yellow-red band. The peak 1 does not shift with increasing $\mathrm{P}_{\mathrm{exc}}$, demonstrates single-exponential decay in TRPL measurements, and has the small value of radiative recombination time $\tau ^{rad}\approx 0.3$ ns at LT. These are important arguments to attribute this peak to recombination in the nonpolar QWs. The peak 2 is lower in energy than the peak 1 and shifts towards the higher energies with increasing $\mathrm{P}_{\mathrm{exc}}$. TRPL reveals non-exponential decay curves of this PL peak with relatively long characteristic times $\tau _{2,3}\geqslant 3$ ns at LT. As stated above, these features are characteristic of radiation from inhomogeneous QWs with a built-in electric field. Given its energy, these are most likely semipolar QWs. Accordingly, the observed decrease of peak 2 intensity in NRs with smaller diameter (A2–C2) is associated with the smaller areas of ($10\bar{1}1$) pyramidal facets in comparison with NRs A1–C1. Interestingly, the peak 2 appears only at sufficiently high pump powers that can be treated as an increase in its radiative recombination rate due to the screening of the built-in field in the QWs. The last component—the yellow-red band–decays on the time-scale of greater than 100 ns and saturates with increasing $\mathrm{P}_{\mathrm{exc}}$ upon above-barrier excitation, being rather weak with under-barrier excitation. These properties are not compatible with QW radiation. We remind also that a similar band was observed in the NRs without QWs. Therefore, we must designate this yellow-red band as predominantly defect-related radiation from GaN.

**Figure 6:**
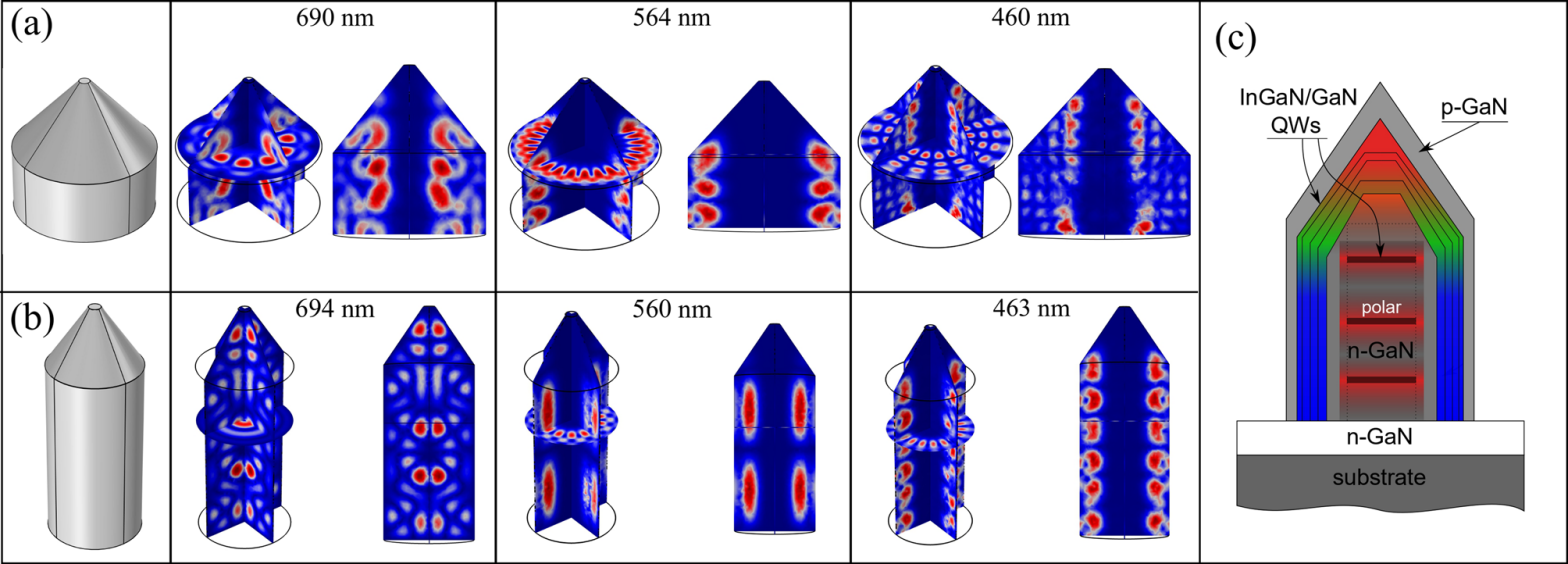
(**a**, **b**) Images of calculated eigenvalues of the electromagnetic field for two geometries at different wavelengths (R, G, B). Geometry (**a**) corresponds to wide NRs (type A1) with a cylinder part with radius $\hbox {r}_1 = 650$ nm, height $\hbox {h}_1 = 600$ nm and a conical part with $\hbox {h}_2=650$ nm. Geometry (**b**) corresponds to narrow NRs (type A2) with a cylinder part with $\hbox {r}_1 = 390$ nm, $\hbox {h}_1 =1500$ nm and a conical part with $\hbox {h}_2=500$ nm. In both cases, upper radius $\hbox {r}_2 = 50$ nm. (**c**) Proposed design of a hybrid NR structure.

We assume that there are two principal factors leading to the weak emission from polar QWs at the tip of the NR. First and the most obvious is the small area of such QWs comparatively with other QWs. This drawback can be partly overcome by growing wide NRs^28^. The second point is the internal electric field of the polar QWs, which decreases a radiative recombination rate. Basically, this rate can be modified (either increased or decreased) by means of the Purcell effect, whose action depends on the density of photon states in a cavity^47^. We have calculated the electromagnetic field distribution inside the structures which are similar to the NRs shown in Fig. 1, i.e. the wide A1 and narrow A2, assuming them as an optical cavity. The calculation details can be found in the Supplementary. Figure 6a,b shows “birds-eye” and side views for eigenmodes at different wavelengths of about 690, 560 and 460 nm. We have chosen these wavelengths for R, G, B because they have the highest Q-factor within these spectral ranges. As one can see in Fig. 6a,b, the electromagnetic field distribution at wavelengths 460 and 560 nm corresponds to whispering gallery modes (WGMs) of a high order $\textit{m}\ge 20$ in the cylindrical part, which should increase the radiative recombination rate in nonpolar QWs in both wide (a) and narrow (b) NRs. On the other hand, the small density of the photon states in the NR tip leads rather to the inhibition of spontaneous recombination in this narrowing volume^47^. Such a situation may be one of the key reasons for the observed inefficient under-barrier optical excitation of the polar and semipolar QWs in the studied NRs.

There is a critical radius $r_{\mathrm{cr}}$ of a NR, at which the transition from the low order WGM to the center mode occurs. For the blue wavelengths, we estimate such a radius as 100-150 nm, and its value increases for bigger wavelengths. Below this value, the electromagnetic field distribution corresponds to the formation of Fabry-Perót modes, which can provide an increase of the spontaneous recombination rate in polar QWs located at field maxima, while this effect does not occur in sidewall QWs. As can be seen in Figure 6b, the electromagnetic field distribution at 690 nm can be concentrated close to the NR axis only in the narrow NRs, which implies the limitation of the NR diameter. It should be noted that in our calculations we used the boundary conditions under which the radiation can be reflected from the base of the NR, as if the NR were split off from the buffer layer. In the as-grown NR samples fabricated on sapphire covered by a GaN buffer, the optical confinement is weaker.

A natural conclusion arising from our experimental and theoretical results is the proposal to place the polar QWs in the core of the NR, as it is shown in Fig. 6c. The area of such QWs will be obviously increased and their number may be taken as needed. This concept is in line with hybrid nanowires for solar cells, comprising coaxial and uniaxial InGaN/GaN QWs^48^. Certainly, the hybrid design does not imply the fabrication of p-n junction for core-located QWs. Pumping of such polar QWs will be by the blue radiation of sidewall nonpolar QWs, similarly to monolithic white LEDs^11,15^. With proper designing, such hybrid structure can act as an efficient converter of the excess of blue luminescence from electrically-pumped sidewall QWs to the red radiation. Manufacture of hybrid structures can involve two stages. First, columns with a set of polar QWs designed for the red spectral range can be made from a planar structure using top-down etching technology. Then, a shell with nonpolar QWs will be grown on the columns. When choosing the column diameter, features of the electromagnetic field distribution should be taken into account.

Summarising, radiative properties of the core–shell NRs with $\hbox {In}_x\hbox {Ga}_{1-x}\hbox {N/GaN}$ QWs grown by MOCVD have been investigated by various spectroscopic techniques. For the NRs with nominal In composition of 12, 19, or 26 %, a pair of PL peaks has been recorded, respectively, in the near-UV, blue, and green spectral ranges. We have shown that these peaks arise from nonpolar and semipolar QWs, while the PL from polar QWs is either absent or hidden in the yellow-red emission related to the defect states in GaN. To clarify the red radiation problem, we have considered the NR as an optical cavity, where the interaction of transitions with optical modes is possible. Calculations of the electromagnetic field distribution inside the NRs of different sizes have shown no chance for the red radiation enhancement, but rather its quenching in conventional NR design. To overcome the red-light problem we propose a hybrid NR design which contains the polar QWs inside the GaN core, pumped by the radiation of nonpolar QWs. We assume that our detailed spectroscopic analysis, which led to the proposal of a hybrid NR design, will be useful in the development of any product from a diverse family of nanorods for optoelectronic and nanophotonic applications.

## Methods

In our studies, we have used the samples with arrays of NRs grown by metal-organic vapor phase epitaxy (MOVPE). The growth procedure was as follows. At first, 750 nm i-GaN and 750 nm n-type GaN buffer layers were grown on a sapphire substrate. Then, a 30 nm thick ${\mathrm{SiO}}_{2}$ mask was fabricated by plasma-enhanced chemical vapor deposition (PECVD) for selective area epitaxy (SAE). An array of holes with two diameters (190 or 460 nm) was created in the ${\mathrm{SiO}}_{2}$ layer by a combination of nanoimprint lithography, photolithography, and dry etching techniques. Afterwards, the templates were loaded in the MOVPE growth chamber and the GaN/InGaN regrowth was carried out in the following sequence: the n-GaN core, three InGaN/GaN QWs and capping p-GaN outer shells (see Fig. S1 in the Supplementary). The growth conditions were similar to those described in a previous paper^28^. The planar template (without a mask) was co-loaded to grow reference polar QWs in the same conditions. The thickness of the wells and barriers in the planar samples were specified by X-ray diffraction as 3-3.5 nm and 15-16 nm, respectively. During the growth of the QWs only the temperature $\mathrm{T}_{\mathrm{gr}}$ was varied to change the wavelength of QW luminescence. The temperature was set to 800, 760 and $720^{\circ }\hbox {C}$ to provide nominal In composition in QWs in the planar samples A, B, and C as 12, 19 and 26 %, respectively (see Table 1). We refer to the NR samples, grown with 460 nm holes in the mask, as A1, B1, and C1, and with 190 nm holes in the mask as A2, B2, and C2. In addition, the reference NR sample without any InGaN QWs (only bare GaN core) was grown.

**Table 1:** Studied planar and nanorod samples.

$\mathrm{T}_{\mathrm{gr}}$, $^{\circ }\hbox {C}$	800	760	720
In content *x* (in planar), %	12	19	26
Planar	A	B	C
NRs (460-nm mask)	A1	B1	C1
NRs (190-nm mask)	A2	B2	C2

NR arrays were examined by SEM using a JEOL JSM-7001F microscope. PL was measured using two setups, either with or without a high spatial resolution. The first setup comprises the LabRAM HR microscope and Horiba Jobin Yvon T64000 triple spectrometer with a liquid nitrogen-cooled CCD. This setup was intended to measure $\mu$-PL of single NRs. For the excitation of PL we used continuous-wave (cw) laser lines with wavelengths of 325 and 405 nm. The second setup was used to measure time-integrated PL and TRPL at temperatures from 10 to 300 K. Incident light was focused on the sample by 18 cm focus lens in the geometry close to back-scattering. PL was guided to a spectrometer (Acton2500i) by two achromatic triplet lenses and detected by either CCD or avalanche photomultiplier module (Becker&Hickl, PMC-150) coupled to a time-correlated single-photon counting system (Becker&Hickl, SPC130). Long-pass filters were used to suppress scattered laser light in the detection path. The temporal resolution of the TRPL setup was about 140 ps. For optical excitation, we used cw-laser lines with a wavelength of 325 and 377 nm, as well as a line from the pulsed laser with a wavelength of 405 nm and frequency of 30 MHz.

Approximation of the peak 1, peak 2, and YL band in the $\mu$-PL spectra of NRs A1, B1, and C1, was carried out using Gaussian functions. At low powers of 0.7 and 7 $\mu \hbox {W}$, the spectra of samples B1 and C1 were fitted with two Gaussian functions corresponding to the peak 1 and YL. For sample A1, the peak 1 has a low energy tail, which has been fitted with an additional Gaussian function. For samples A1 and B1, the fitted values of peak energy ($\hbox {E}_{\mathrm{max}}$) and FWHM of the YL band at $\mathrm{P}_{\mathrm{exc}}=0.7$ $\mu \hbox {W}$ are $2.21\pm 0.01$ eV and $460\pm 10$ meV, respectively. For sample C1, the peak 1 and YL band are merged and the spectrum approximation problem has no unambiguous solution. For this sample, we used the same values of $\hbox {E}_{\mathrm{max}}$ and FWHM for the fitting of the YL band, as for samples A1 and B1, since the YL band does almost not depend on the sample, which makes it possible to approximate the peak 1 in the sample C1 at 0.7 $\mu \hbox {W}$. At the higher powers, the spectral width and position of the Gaussian functions describing the peak 1 and YL were used nearly the same as at 7 $\mu \hbox {W}$, while the function describing the peak 2 was fitted without restrictions.

## Supplementary information

Supplementary Information.
