# Supplementary material for: State-of-the-art and prospects for intense red radiation from core–shell InGaN/GaN nanorods
Source: Sci Rep. 2020 Nov 4;10:19048. doi: 10.1038/s41598-020-76042-0 (PMC7643183; doi:10.1038/s41598-020-76042-0)
Supplement: Supplementary file 1 — Supplementary Information. [file 41598_2020_76042_MOESM1_ESM.pdf]

Supplementary information for

"State-of-the-art and prospects for intense red radiation from core-shell InGaN/GaN nanorods"

Evgenii A. Evropeitsev[1,]*, Dmitrii R. Kazanov[1], Yoann Robin[2], Alexandr N. Smirnov[1], Ilya A. Eliseyev[1], Valery Y. Davydov[1], Alexey A. Toropov[1], Shugo Nitta[2], Tatiana V. Shubina[1], and Hiroshi Amano[2]

1: Ioffe Institute, 26 Politekhnicheskaya, St Petersburg 194021, Russia

2: Institute of Materials and Systems for Sustainability (IMaSS), Nagoya University, Nagoya, Japan

* evropeitsev@beam.ioffe.ru

I. Samples growth

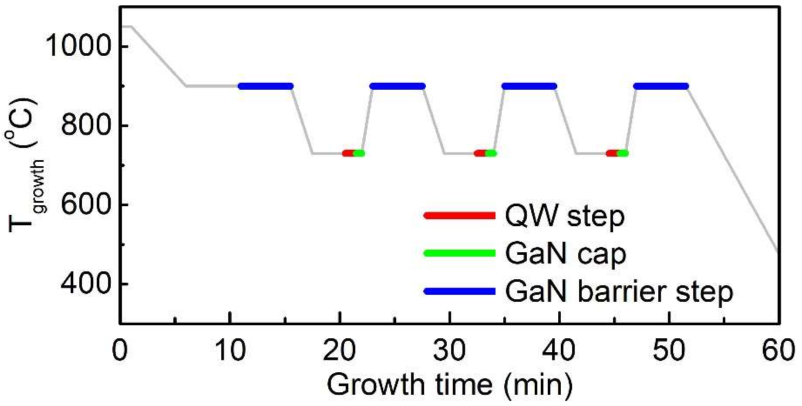

Figure S1. Growth sequence of MOVPE for InGaN/GaN QWs in a shell of nanorods.

II. Power-dependent µ-PL in the NRs

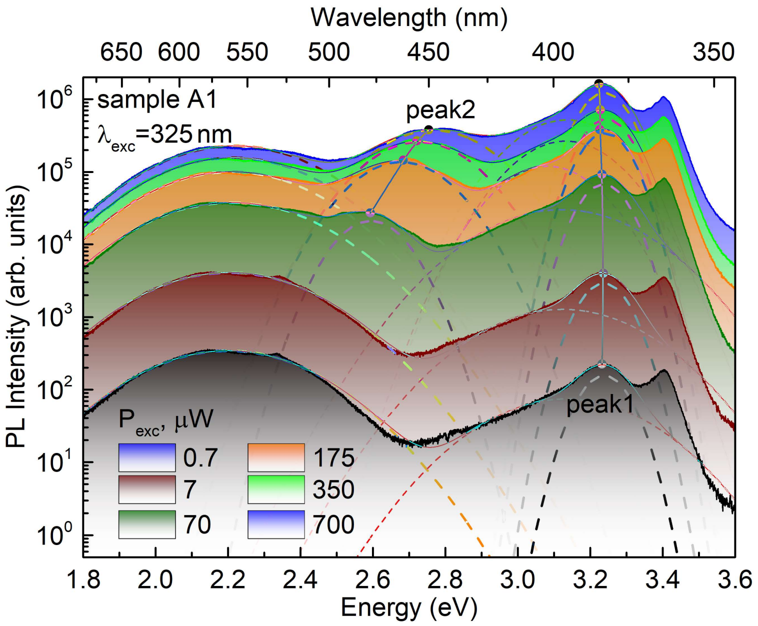

Figure S2. Power-dependent µ-PL spectra measured at room temperature (RT) in the NR sample A1 excited by a 325-nm cw-laser line.

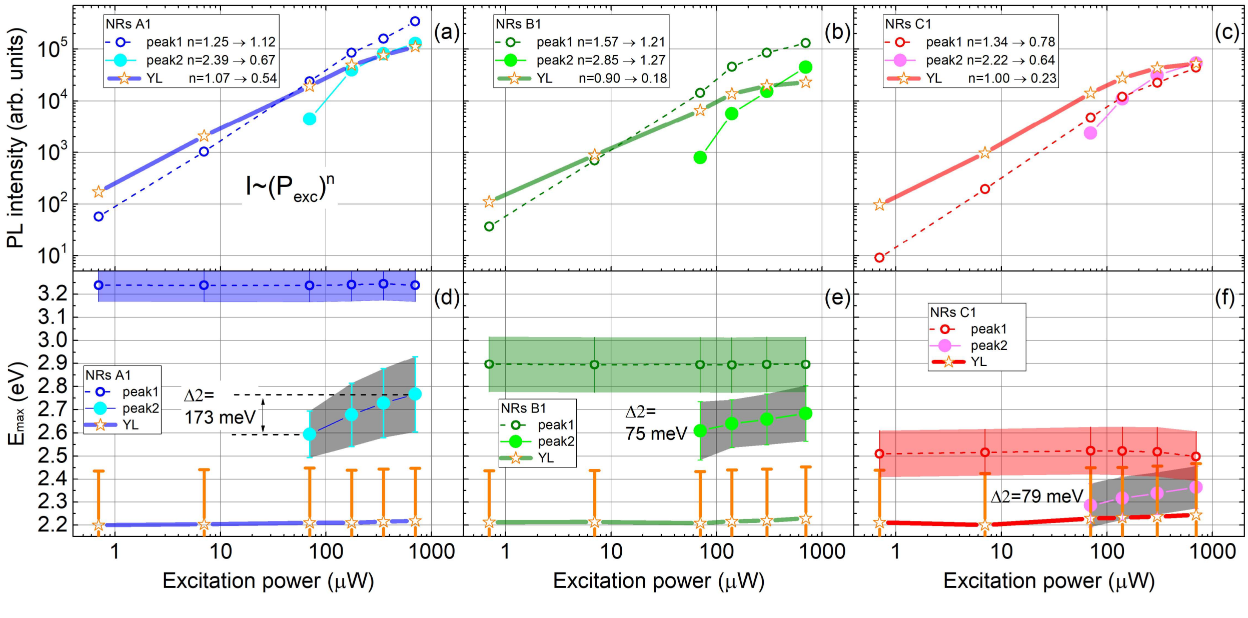

Figure S3. Dependence of µ-PL intensity (a, b, c) and peak energy (d, e, f) of peak 1, peak 2, and the defect-related yellow line (YL) in the NRs A1, B1, and C1, respectively, on the excitation power (P_{exc}). In the legend of figures (a-c), the values of the coefficient n in the formula $I \sim (P_{exc})^n$ are shown, which was extracted from the power dependence of the integral intensity of PL for the two minimum and two maximum values of P_{exc}. Fitting of the original spectra was done by Gaussian functions. Bars in (d-f) show FWHM of the Gaussian functions. The µ-PL spectra were measured at RT, with excitation by a 325 nm cw-laser line.

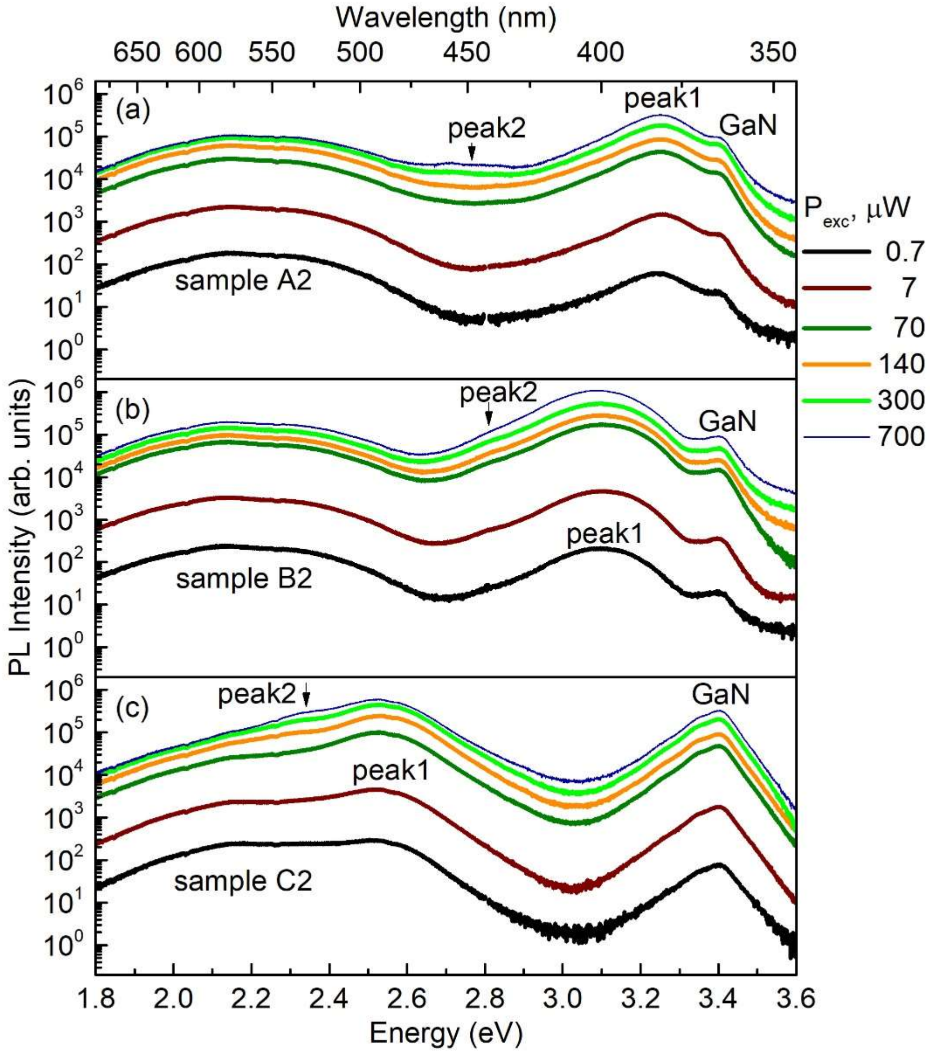

Figure S4. Power-dependent μ-PL spectra measured at RT in the NRs samples A2, B2, and C2 excited by a 325-nm cw-laser line.

Figure S4 shows the μ-PL spectra of the NRs series A2-C2. As can be seen, the peak°2 occurs only at maximum excitation powers, and it is weaker than that in the spectra of the nanorods A1-C1 with a larger diameter, which are shown in Fig. S2 and Fig. 3 of the main text. This fact indicates the origin of the peak°2 from the semipolar or polar QWs, the areas of which strongly decrease in the NRs of the A2-C2 series.

III. PL in the planar samples

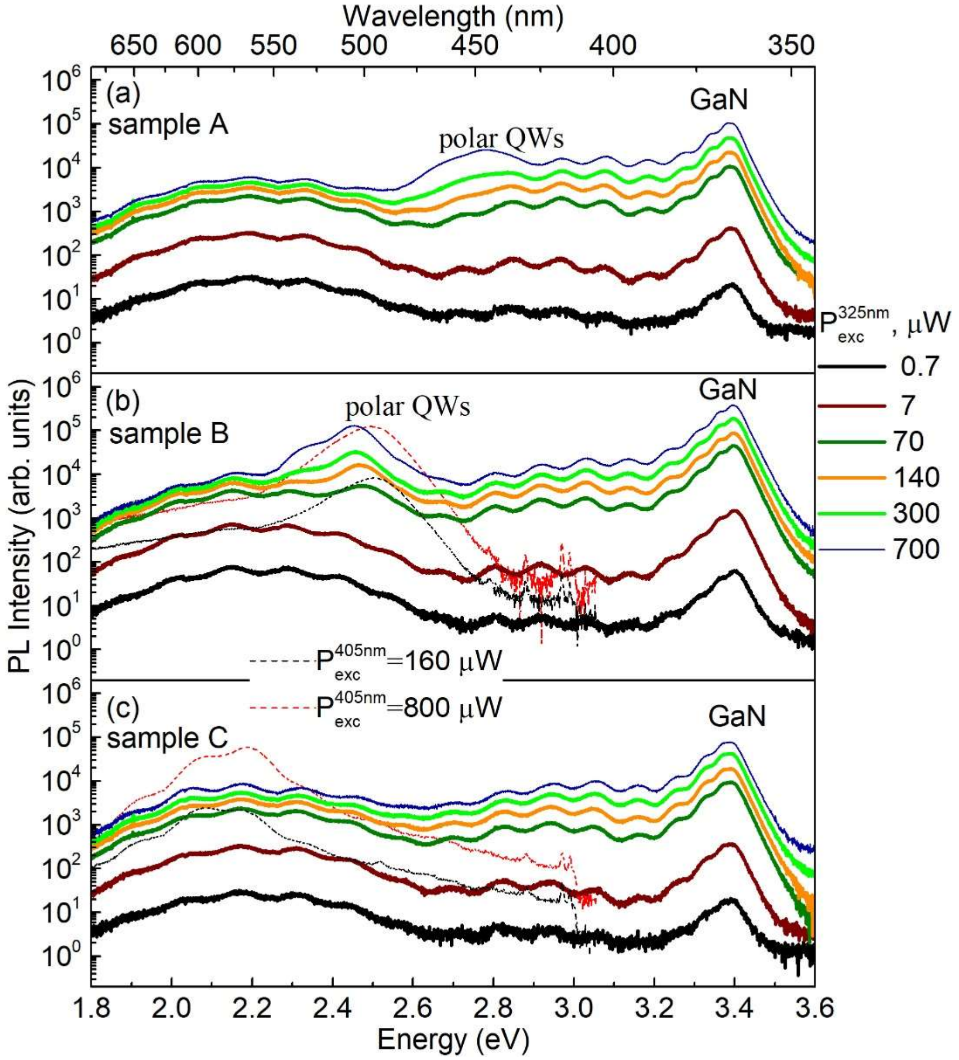

Figure S5. Power-dependent μ-PL spectra measured at RT in the planar samples A, B, and C excited by a cw-laser line with a wavelength of 325 nm. The PL spectra excited by the 405 nm line are shown by dashed lines in (b) and (c). The positions of the peak at the under-barrier excitation allow us to indicate the QW emission.

Figure S5 shows the spectra of μ-PL measured in planar samples A, B, and C at RT. The spectra are modulated by an interference pattern of Fabry-Perot modes. The corresponding spectral positions of QW-related PL are at a wavelength of about 450, 505, and 570 nm, respectively. With the above-barrier excitation (λ_{exc} = 325 nm), QW-related PL in samples A and B appears only at sufficiently high P_{exc}. At low P_{exc}, emission at the absorption edge (near-band-edge emission, NBE) and the yellow luminescence (YL) band associated with defects prevail in the spectra. PL of sample C dominates by broad defect bands and NBE in the all used range of P_{exc} (Figure S5 (c)). On the contrary, in the case of quasi-resonant (under-barrier) excitation, the QW-related PL is visible even at a relatively small P_{exc} (Figure S5 (b, c), dashed lines).

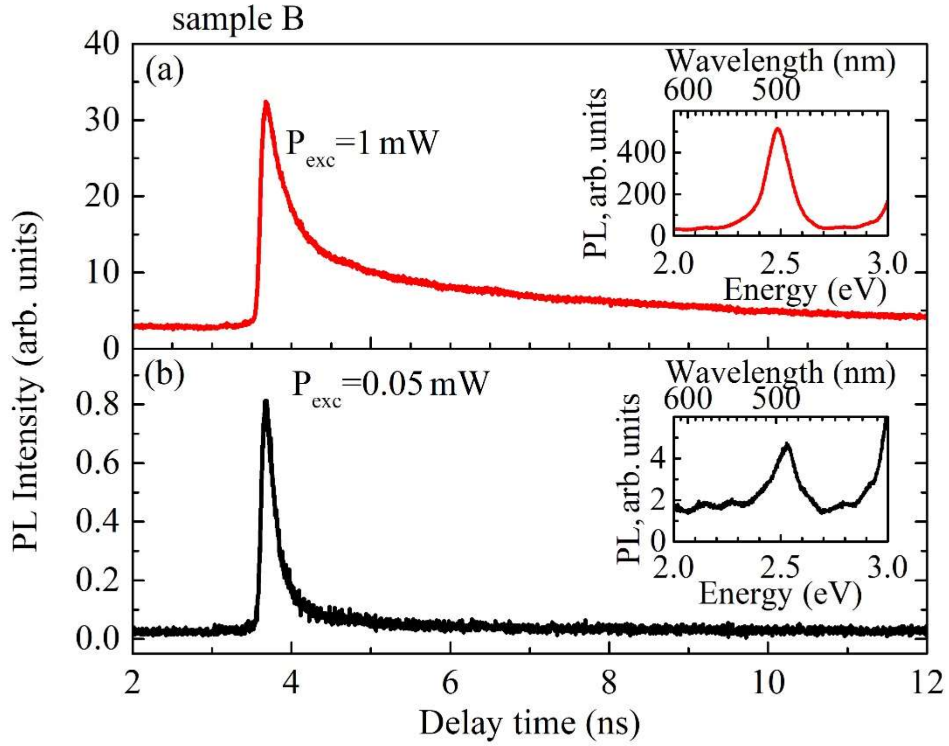

Figure S6. PL decay curves measured in planar sample B excited by a 405-nm laser line with an average power of 1 mW (a) and 0.05 mW (b) and a repetition rate of 30~MHz. The detection wavelength is 500 nm. Insertions show corresponding PL spectra.

At the low temperatures (LT), TRPL measurements showed decay times of 50-150 ns in the planar QWs. Such a slow PL decay is commonly explained by a small e-h overlap because of the presence of an electric field across the InGaN/GaN polar QWs, which induces the QCSE. At RT, the PL decay is characterized by a sub-nanosecond decay time, the value of which depends on the P_{exc}. Figure S5 shows the PL decay curves measured in the planar sample B with two different excitation powers – 1 mW (a) and 0.05 mW (b). At the higher P_{exc}, the PL decay time is longer (~0.8 ns), while it is shorter (~0.16 ns) at the smaller P_{exc}. We explain such variation by the fact that the decay time is controlled by a fast nonradiative recombination channel which dominates at the smaller P_{exc}. The saturation of nonradiative centers at the higher P_{exc} leads to the increase in decay time. Internal quantum efficiency (IQE) of PL in sample B at a wavelength of 500 nm, evaluated as a ratio I(RT)/I(LT), is 0.28% (λ_{exc}=377 nm, P_{exc}=15 mW), where I is the integral PL intensity.

IV. Calculation of the electromagnetic field distribution

We consider the NR as a microcavity, where the value of the radiative recombination rate can be increased or decreased due to the Purcell effect under conditions of weak coupling between the confined optical mode and optical transitions inside the cavity. It works by analogy with the Fermi's "golden rule" and depends on the density of photon states and the correspondence of the frequencies of optical and material resonances [1]. A possible modification of the radiative recombination rate due to the Purcell effect for a single cavity mode (wavelength λ) can be expressed as [2]

$$\frac{\tau^{rad}(\lambda)}{\tau} = \frac{2}{3} F_p \frac{|E(r)|^2}{|E_{max}|^2} \frac{\Delta\lambda^2}{\Delta\lambda^2 + 4(\lambda - \lambda_{exc})^2}, \qquad (1)$$

where τ^{rad} and τ are the intrinsic and changed radiative recombination times, respectively, $\Delta\lambda$ is the spectral width of the resonant mode, E(r) is the electromagnetic field distribution, F_P is the Purcell factor that depends on the characteristics of the resonator [3]

$$F_P = \frac{3}{4\pi^2} \left(\frac{\lambda}{n}\right)^3 \frac{Q}{V_{eff}}, \qquad (2)$$

where $Q = \lambda/\Delta\lambda$ is the quality factor of the mode, n is the refractive index of the medium and V_{eff} is the effective mode volume.

The photon density of states can be visualized by calculating the electromagnetic field distribution in a cavity of a certain geometry. As it was previously reported, such a distribution can be complex and inhomogeneous in space inside a monolithic microcavity [4-6]. An increase in the radiative radiation rate ($1/\tau$) allows observing the increase in radiation intensity. On the other hand, the intensity can quench when the photon density is low, e.g., when a cavity size is less than a radiation wavelength [1].

We have studied the electromagnetic field distribution inside the structures which are similar to the NRs A1 and A2. The simulations were implemented via the finite-difference time-domain (FDTD) method using Comsol Multiphysics software. We replace the hexagonal structure by a cylindrical one, which does not drastically influence the mode composition [7]. The real size cavity was located inside the large cylinder "box" of air with scattering boundaries. This boundary condition allowed us to avoid scattering waves from the "box", which could create additional interference patterns. The refractive index was chosen to be

$$n_{mean} = (n_1 V_1 + n_2 V_2)/\,(V_1 + V_2), \qquad (3)$$

where n_1 – refractive index of GaN and n_2 – refractive index of $In_{0.15}Ga_{0.85}N$; V_1 and V_2 are respective volumes. However, this value is very close to n_1 because of the small volume of QWs.

References

[1] Kleppner, D. Inhibited spontaneous emission, *Phys. Rev. Lett.* **47**, 233 (1981).

[2] Gerard, J. M. *et al.* Enhanced spontaneous emission by quantum boxes in a monolithic optical microcavity. *Phys. Rev. Lett.* **81**, 1110 (1998)

[3] Purcell, E. M. Spontaneous emission probabilities at radio frequencies. *Phys. Rev.* **69**, 681 (1946).

[4] Shubina, T. V. *et al.* III-nitride tunable cup-cavities supporting quasi whispering gallery modes from ultraviolet to infrared, *Sci. Rep.* **5**, 17970 (2015).

[5] Shubina, T. V. *et al.* III-nitride microcrystal cavities with quasi whispering gallery modes grown by molecular beam epitaxy. *Phys. Status Solidi B*, **1**, 1 (2016).

[6] Kazanov, D. R., Evropeytsev, E. A. & Shubina, T. V. The cavity-effect in site-controlled GaN nanocolumns with InGaN insertions. *Semiconductors* **53**, 2085 (2019).

[7] Nobis, T., Kaidashev, E. M., Rahm, A., Lorenz M. & Grundmann, M. Whispering Gallery Modes in Nanosized Dielectric Resonators with Hexagonal Cross Section. *Phys. Rev. Lett.* **93**, 103903 (2004).
